# Intracranial hypotension caused by cisternal irrigation for vasospasm after subarachnoid hemorrhage: a case report

**DOI:** 10.1186/1752-1947-8-308

**Published:** 2014-09-15

**Authors:** Atsushi Ishida, Seigo Matsuo

**Affiliations:** 1 Department of Neurosurgery, Moriyama Memorial Hospital, 7-12-7 Nishikasai, Edogawa-ku, Tokyo 134-8608, Japan

**Keywords:** Vasospasm, Cisternal irrigation, Brain sagging

## Abstract

**Introduction:**

Vasospasm is the most common cause of complication after a subarachnoid hemorrhage and tremendous efforts have been made to prevent it. A subarachnoid clot is the cause of the vasospasm and dissolving and washing it out is considered to be the best practice. Cisternal irrigation with urokinase and ascorbic acid has been widely used due to its proven effect.

**Case presentation:**

A 60-year-old Japanese male presented with a severe headache was diagnosed with a subarachnoid hemorrhage and an immediate surgical obliteration was successfully performed. After clipping the aneurysm, a cisternal drainage tube was placed in the chiasmatic cistern. In order to clear the thick subarachnoid hemorrhage, a cisternal irrigation was performed. However, his consciousness deteriorated and his left pupil became dilated on the next day. A T1 sagittal magnetic resonance imaging scan showed an evidence of marked brain sagging with mild tonsillar descent. We continued intensive hydration and head-down positioning and the brain sagging was shown to have improved in the follow-up magnetic resonance imaging scan.

**Conclusions:**

We present a case in which our patient experienced brain sagging after a cisternal irrigation of a subarachnoid hemorrhage. A subdural hematoma and low intracranial pressure suggested intracranial hypotension. Sagittal magnetic resonance imaging images are useful to evaluate brain sagging and are shown sequentially here in our case report.

## Introduction

Symptomatic vasospasm is one of the most hazardous problems affecting morbidity and mortality after an aneurismal subarachnoid hemorrhage (SAH) [1]. It is now generally considered that cerebral vasospasm is induced by some spasmogenic substances produced by a clot around the cerebral arteries. Cisternal irrigation therapy with urokinase (UK) has been performed to prevent symptomatic vasospasm by dissolving and removing SAH, and excellent results have been obtained [2,3]. In our case report, cisternal irrigation therapy with urokinase (UK) resolved an extremely thick SAH and our patient did not suffer from symptomatic vasospasm. However, soon after the irrigation, his level of consciousness deteriorated caused by brain sagging due to intracranial hypotension. To the best of our knowledge, this is the first case of intracranial hypotension caused by cisternal irrigation.

## Case presentation

**Figure 1:**
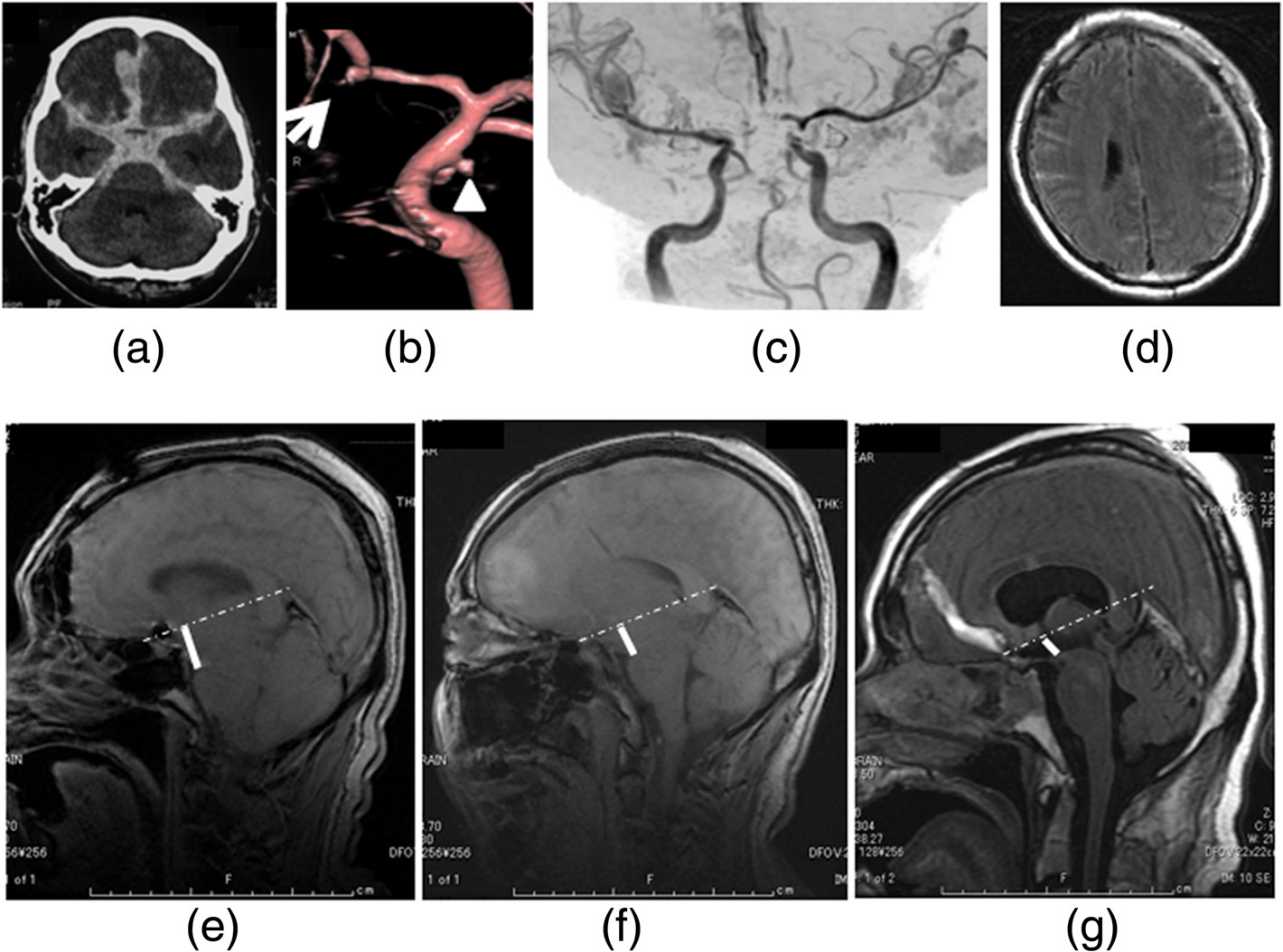
**Clinical course of the case. (a)** Computed tomography (CT) scan showing diffuse, thick SAH and hematoma in the right frontal lobe. **(b)** DSA showing a left IC-PC aneurysm (arrowhead) and another suspected aneurysm at A-com (arrow). **(c)** MRA showing no severe vasospasm. **(d)** MRI showing fresh subdural hematoma **(e)** T1 sagittal MRI (day 4) showing marked brain sagging with mild tonsillar descent. A dotted straight line is drawn from the tuberculum sellae to the confluence of the straight sinus. A thick line is drawn indicating the distance from the mammillary body to the line. **(f)** T1 sagittal MRI (day 15) showing the shorter distance than that of day 4. **(g)** T1 sagittal MRI (day 21) showing the shorter distance than that of day 15. SAH, subarachnoid hemorrhage; DSA, digital subtraction angiography; IC-PC, internal carotid artery-posterior communicating artery; MRA, magnetic resonance angiography; MRI, magnetic resonance image.

A 60-year-old Japanese male presented with severe headache and was transferred to our hospital 12 hours after the onset of symptoms. Diffuse, thick SAH was observed on his computed tomography (CT) scan and a hematoma in the right frontal lobe suggested a ruptured aneurysm at the distal anterior cerebral artery (ACA) or anterior communicating (A-com) artery (Figure 1a). The degree of SAH of the patient was classified as Fisher CT Group 3, and the highest CT number (Hounsfield number) exceeded 60 in the SAH, which suggested a significant risk for symptomatic vasospasm (Figure 1a). Subsequent digital subtraction angiography (DSA) showed the same information on the aneurysms and showed no aneurysm at the distal ACA (Figure 1b). An immediate operation was considered the best course of action and a left front-temporal craniotomy was chosen in order to reach both aneurysms. After the introduction of general anesthesia, we inserted a spinal drainage tube which was kept closed until the craniotomy was completed. There was a left internal carotid artery (IC)-posterior communicating (PC) aneurysm which was obviously not the rupture origin, but it was obliterated as well. After clipping the aneurysm, we followed the left A1 and found the A-com aneurysm. This appeared to be the rupture origin and a complete neck clipping was performed without event.

After clipping the aneurysm, a cisternal drainage tube was placed in the chiasmatic cistern. Two days after the operation (day 2), 500ml of Lactated Ringer's solution with urokinase (120IU/mL), and ascorbic acid (4mg/mL) was infused at a rate of 30mL/hour from the cisternal drainage tube and drained out from the lumber drainage set at the level of the external auditory canal. We carefully checked his level of consciousness and also performed the same procedure the next day (day 3). However, his consciousness deteriorated and his left pupil became dilated on the next day (day 4), despite the fact that his magnetic resonance imaging (MRI) scan showed no severe vasospasm (Figure 1c). His MRI showed a subdural hematoma (Figure 1d) which was not present previously. His intracranial pressure level was below the level of the external auditory canal (EAC). The deterioration of his level of consciousness might have been caused by brain sagging due to intracranial hypotension. His T1 sagittal MRI showed evidence of marked brain sagging with mild tonsillar descent (Figure 1e). The quantification of brain sag has been described by measurement of distance between a straight line drawn from the tuberculum sellae to the confluence of the straight sinus and the mammillary body in a sagittal view of MRI. The mammillary body normally lies on this line [4]. Both of his pupils became dilated on day 6. Even though his MRA (magnetic resonance angiography) showed no angiographic vasospasm, his level of consciousness did not improve. We continued intensive hydration and head-down positioning and the brain sagging improved in his follow-up MRI scans (day 15 and 21) (Figure 1f, g). Those MRI images showed a gradual enlargement of the ventricles and worsening of the periventricular lesion (PVL). A lumber puncture was performed on day 23 and the initial pressure was 40cmH_2_O. A ventriculoperitoneal (VP) shunting was performed, after which he dramatically improved.

## Discussion

Fisher *et al*. [2] reported that vasospasm was found in almost all cases with a thick and widespread subarachnoid clot on a CT scan (group 3 according to their grading scale). That is because some spasmogenic substances produced from a clot around the cerebral arteries cause cerebral vasospasm. Cisternal irrigation therapy with urokinase (UK) has been performed to prevent symptomatic vasospasm by dissolving and removing SAH [2,3]. Kodama *et al*. reported that complications occurred in eight patients during irrigation therapy; two patients experienced seizures, two patients developed meningitis, and four patients had an intracranial hemorrhage. However, all of these patients recovered without neurological deficits [3].

In this case, the SAH was very thick and a severe vasospasm was easily predicted to occur. We followed the protocol used in the previous report [3] and the SAH was washed out dramatically without the complications described. However, brain sagging occurred soon after the irrigation. We followed the same protocol as previous irrigations but have never experienced this complication. Intracranial hypotension is also associated with brain sag [4]. A retrospective study found that 48% of patients with intracranial hypotension also had descent of the brain [5]. Patients can have slit ventricles, distortion of the brain stem, and cerebellar tonsillar herniation [6]. Brain sagging can result in transtentorial descent of the diencephalon, potentially leading to changes in consciousness [7]. In this case, the distance was the widest soon after the irrigation (Figure 1e) and was gradually reducing (Figure 1f, g). As his intracranial hypotension improved, his CSF pressure elevated and he started to suffer from hydrocephalus. Surprisingly, the pressure increased to 40cmH_2_O. We hypothesize that the malabsorption of cerebrospinal fluid (CSF) usually seen after SAH became apparent as the intracranial hypotension was resolved.

Treatment options include conservative medical therapy, epidural or intrathecal injections, and/or surgery. Medical therapy involves bed rest, oral hydration, caffeine, and/or steroids [8]. Hydration aims to increase CSF volume. The most commonly reported treatment for intracranial hypotension is an epidural blood patch (injecting autologous blood into the spinal epidural space) [9]. In this case, we inserted the spinal drainage tube on the first attempt, and there was no leakage around the tube. After the tube was removed, his lumber spinal MRI scan showed no CSF leakage. Therefore, we did not attempt a blood patch, and focused on hydration and head-down positioning. The brain sagging recovered without the use of a blood patch.

## Conclusions

Cisternal irrigation therapy with UK is an excellent procedure to prevent vasospasm after SAH. However, it can cause intracranial hypotension and brain sagging which can result in transtentorial descent of the diencephalon, leading to changes in consciousness. Though it seems very devastating, the condition can be reversible with intensive treatment such as hydration.

## Consent

Written informed consent was obtained from the patient for publication of this case report and accompanying images. A copy of the written consent is available for review by the Editor-in-Chief of this journal.

## Abbreviations

SAH: subarachnoid hemorrhage; UK: urokinase; CT: computed tomography; ACA: anterior cerebral artery; A-com: anterior communicating; DSA: digital subtraction angiography; IC-PC: internal carotid-posterior communicating; EAC: external auditory canal; VP: ventriculoperitoneal; CSF: cerebrospinal fluid.

## Competing interests

The authors declare that there are no competing interests.

## Authors’ contributions

AI analyzed and interpreted the patient data, wrote the case report and revised the manuscript. SM supervised for the case. Both authors read and approved the final manuscript.
